# Factors influencing uptake, continuation, and discontinuation of oral PrEP among clients at sex worker and MSM facilities in South Africa

**DOI:** 10.1371/journal.pone.0228620

**Published:** 2020-04-30

**Authors:** Diantha Pillay, Kayla Stankevitz, Michele Lanham, Kathleen Ridgeway, Mercy Murire, Elmari Briedenhann, Sarah Jenkins, Hasina Subedar, Theresa Hoke, Saiqa Mullick

**Affiliations:** 1 Wits Reproductive Health and HIV Institute, Faculty of Health Sciences, University of the Witwatersrand, Johannesburg, South Africa; 2 FHI 360, Durham, North Carolina, United States of America; 3 Clinton Health Access Initiative, Pretoria, South Africa; 4 National Department of Health, Pretoria, South Africa; University of South Florida, UNITED STATES

## Abstract

**Background:**

South Africa became the first country in Africa to introduce oral PrEP in June 2016. The National Department of Health has used a phased approach to rollout, allowing for a dynamic learn-and-adapt process which will lead ultimately to scale-up. Phased rollout began with provision of oral PrEP at facilities providing services to sex workers in 2016 and was expanded in 2017, first to facilities providing services to MSM and then to students at selected university campus clinics, followed by provision at primary health care facilities. Programmatic data shows variability in initiation and continuation between these populations. This study examines factors related to PrEP initiation, continuation, and discontinuation at facilities providing services to sex workers and MSM during the national PrEP rollout.

**Methods:**

A cross-sectional survey was administered September 2017-January 2018 among clients (ages 18–62 and providers at 9 facilities implementing oral PrEP in South Africa, followed by in-depth interviews. The client survey captured PrEP initiation, continuation and discontinuation. Analysis was performed in STATA 13 for survey data and thematic analysis was performed in NViVO 11 for in-depth interview data.

**Results:**

299 clients (203 from sex worker facilities, 96 from MSM facilities) participated in the survey and additionally, in-depth interviews were conducted with 29 clients. Participants self-identified as either current users (n = 94; 36.2%), past users (n = 80; 30.8%) and never users of PrEP (n = 86; 33.1%). Participants who had never used PrEP either cited not being offered PrEP by a provider (57%, n = 49) or declining PrEP (43%, n = 37) as reasons for lack of uptake. The primary reason for declining to use oral PrEP was fear of side effects (41.7%, n = 15). The primary reasons for initiating and continuing on oral PrEP were all related to perceived risk associated with sexual activity. The majority of participants (87.9%, n = 153) also noted that printed IEC materials influenced their decision to initiate PrEP. Qualitative data suggested that several clients initiated on PrEP because they wanted additional protection beyond using condoms due to challenges such as partners refusing to use condoms, having partners with unknown HIV status, having multiple partners, involvement in sex work, or having a partner living with HIV. The majority (73.8%, n = 59) of participants who discontinued oral PrEP cited side effects as the primary reason for discontinuation, followed by feeling stigmatized (18.8%, n = 15).

**Conclusion:**

This study provides valuable insights on early rollout of PrEP of how clients perceive oral PrEP and where to target efforts to improve the uptake of this highly effective HIV prevention product. By identifying strengths and areas for improvement, the ACCESS study has generated evidence that can be used to guide high quality scale-up in South Africa and may be instructive for other countries’ efforts to expand quality access to oral PrEP.

## Introduction

Oral pre-exposure prophylaxis (PrEP) for HIV, which is a combination of the antiretroviral (ARV) tenofovir disoproxil fumarate (TDF) and emtricitabine (FTC), has been shown to be effective in reducing the risk of HIV acquisition in clinical trials in a wide variety of settings and populations [1]. Acting on this evidence, the World Health Organization (WHO) published an early-release of its new guidelines on the use of ARV-based prevention products (along with guidelines on early treatment of HIV infection) in September 2015 (updated in June 2016) [2]. These newer guidelines recommend that people with a substantial risk of HIV infection should be provided with daily oral PrEP as part of a combination HIV prevention strategy. “Substantial risk” as defined by the WHO in these guidelines is as follows: *Substantial risk of HIV infection is provisionally defined as an incidence of HIV higher than 3 per 100 person-years in the absence of pre-exposure prophylaxis (PrEP)*.

South Africa has the largest HIV epidemic in the world. With 7 million people living with HIV, the country accounts for 19% of the global burden of HIV, 15% of new HIV infections, and 11% of AIDS-related deaths [3]. Sex workers, men who have sex with men (MSM), and adolescent girls and young women (AGYW) are among the most affected populations [4]. HIV prevalence among female sex workers (SWs) in South Africa is estimated to be 59.8% [5]. In population-based studies among South African MSM, HIV prevalence ranged from 8.6% to 49.5%; an estimated 9.2% of the country’s new HIV infections are in MSM [6]. Thirty-seven percent of new HIV infections in South Africa are in AGYW ages 15 to 24 [4].

In response to the updated WHO guidelines, South Africa became the first country in Africa to register and provide oral PrEP for HIV prevention in June 2016. The National Department of Health (NDoH) is using a phased approach to rollout, allowing for a dynamic learn-and-adapt process which will lead ultimately to scale-up. Phased rollout began with provision of oral PrEP at facilities providing services to sex workers in 2016 and was expanded in 2017, first to facilities providing services to MSM and then to students at selected university campus clinics. Provision of oral PrEP to AGYW at primary health care facilities began in early 2018.

*We Are the Generation that Will End HIV* (WATG), was a slogan used in all NDoH information, education and communication (IEC) and social mobilization materials, which were used to market PrEP as an additional HIV prevention option. A range of IEC materials for clients were developed, including posters, a frequently asked questions brochure, a fact sheet, and a pocket-sized booklet about initiating PrEP. The IEC materials were developed with input from multiple stakeholders, including potential recipients, and adapted for each key population. The content and slogan remained unchanged, but creative elements were adapted according to population preferences. These materials were used to raise awareness and assist facilities with efforts to create demand for oral PrEP.

The NDoH has highlighted that both initiation and continuation of oral PrEP at sex worker (SW) clinics remain lower than anticipated [7]. At facilities providing PrEP services to sex workers from June 2016 to December 2018, 87% of the nearly 71,000 HIV tests conducted were negative [8]. Sixty-nine percent of those who tested negative were offered oral PrEP, and 15% of those who were offered it initiated oral PrEP [8]. Likewise, more than 17,500 HIV tests were conducted from April 2017 to December 2018 at facilities providing services to MSM, of which 96% were negative [8]. Thirty-one percent of MSM who tested negative were offered oral PrEP, and 45% of those clients initiated it [8].

Understanding initiation and continuation of oral PrEP is a priority for the NDoH. Monitoring data allows for tracking program outputs but cannot provide detailed information about the factors that may influence these trends. At the request of the NDoH, the OPTIONS (Optimizing Prevention Technology Introduction on Schedule) Consortium, the HIV Prevention Market Manager (PMM) project, and the NDoH collaborated to carry out operations research under the ACCESS study (Advancing PrEP: Comprehensive and Combined Evaluation of Services for Sex workers and men who have sex with men [MSM]). OPTIONS is a 5-year USAID funded technical assistance grant aimed to provide targeted support to expedite and sustain access to ARV-based HIV prevention products in countries and among populations where most needed. Core partners include Wits RHI, AVAC and FHI 360 and primary partners include Avenir Health, FSG, LVCT Health, LSHTM, McCann Health, and Pangaea Zimbabwe AIDS Trust. The HIV Prevention Market Manager (PMM) Project is a Bill and Melinda Gates Foundation-supported grant through which AVAC and CHAI seek to facilitate an efficient and effective rollout of HIV prevention products. The PMM works with partners across the prevention research to rollout spectrum to expand the portfolio of options and ensure appropriate products are available, accessible and used by those who need them most. ACCESS aimed to identify factors influencing oral PrEP uptake and continuation and to gain a better understanding of clients’ experiences with oral PrEP services. The results are intended to inform further strengthening and rollout of oral PrEP services.

## Materials and methods

### Study setting

The study was set in facilities that were providing oral PrEP as part of the package of HIV prevention services. At the time of the study, oral PrEP had been rolled out at facilities providing services to SWs and MSM (referred to as SW and MSM sites). SW and MSM sites refers to clinics that provide services to key populations. Even though these clinics provide services to key populations (sex workers, MSM), clients who do not identify as either sex workers or MSM (such as but not limited to truck drivers or clients of sex workers) can still access services from these clinics.

### Study objectives

The goal of this study was to identify barriers and facilitators to oral PrEP uptake, retention, and adherence in South Africa. The study had three objectives: (1) to examine factors affecting clients’ decision to initiate and continue or stop oral PrEP use, including perceptions of risk and side effects; (2) to assess provider knowledge, attitudes, and practiced behaviours around oral PrEP delivery; and (3) to assess oral PrEP IEC materials. Objectives 1 and 3 will be presented in this paper.

### Study design

This cross-sectional observational study applied mixed methods: a quantitative survey and follow-up in-depth interviews. The COREQ criteria for reporting qualitative research are used to describe qualitative data collection and analysis [9] and COREQ checklist completed (S1 Appendix).

#### Facility eligibility and selection

Facilities were purposively selected in collaboration with NDoH from among the 16 facilities that were providing oral PrEP at study launch. To include a diverse range of clinics in the sample, we selected facilities based on four main criteria. Clinics were: (1) providing oral PrEP for a minimum of three months; (2) offering different service delivery models (fixed clinic and mobile services); (3) in a variety of locations (rural, urban, and peri-urban); and (4) having varying rates of oral PrEP uptake (by ~2% to ~50% of individuals with negative HIV tests) based on routine data at the time. The nine facilities selected are shown in Table 1.

**Table 1:** Descriptions and locations of sites providing PrEP, May 2017.

Site	Primary population served	Province	Location	Delivery model	PrEP uptake^1^
Site 1	SW	Limpopo	Rural^2^	Fixed	6%
Site 2	SW	Limpopo	Rural	Fixed	2%
Site 3	SW	Gauteng	Peri-urban^3^	Fixed	8%
Site 4	SW	KwaZulu-Natal	Urban^4^	Mobile	10%
Site 5	SW	KwaZulu-Natal	Urban	Fixed	49%
Site 6	SW	Gauteng	Urban	Mobile	22%
Site 7	MSM	Gauteng	Urban	Fixed	10%
Site 8	MSM	Western Cape	Urban	Fixed	13%
Site 9	MSM	Gauteng	Urban	Fixed	25%

^1^Percentages are from routine statistics on PrEP uptake provided by NDoH from June 2016 to May 2017 based on the formula of PrEP uptake = initiated PrEP/offered PrEP x 100

^2^ rural refers to location that is not near a town or city and generally not as well developed or resourced

^3^ peri-urban is a term commonly used in Africa, and refers to a location adjacent to a city or town

^4^ urban refers to a city or town

#### Study population

The study population was HIV-negative men and women ages 18 years and older who were accessing services through the nine selected sites. This population included but was not limited to sex workers, clients of sex workers, partners of sex workers, truckers, MSM, and any other individuals from the surrounding communities attending the facilities.

#### Sample size calculations

The minimum sample size for the survey was calculated based on retrospective site-level data on the number of oral PrEP initiations from June 2016 to May 2017. Retrospective site-level data provided us with information on absolute numbers of clients on PrEP at the selected sites, which allowed us to determine a minimum number of clients we expected to currently be using PrEP. Therefore, a minimum sample of 30 participants per site was proposed (10 current users, 10 past users and 10 never users), with a minimum total of 270 participants across nine sites. An assumption was made that an equal distribution of current, past, and never users of oral PrEP would be reached within the total minimum sample. Current users were defined as clients using oral PrEP at the time of recruitment into the study. Past users were defined as clients who had used oral PrEP previously but were not using oral PrEP at the time of recruitment, which could include clients who had discontinued use or those who might be cycling on and off oral PrEP. Never users were defined as clients who had never used oral PrEP.

### Survey data collection

#### Recruitment and eligibility screening

Survey participants were recruited in September and October 2017 during clinic visits by fieldworkers from a private research firm who approached clients in clinic waiting areas. With the assistance of facility staff, the fieldworkers were directed to current, past, and never users of PrEP. Clients were eligible to participate if they were HIV negative, age 18 or older, and accessing services from the selected facilities (see Table 1). The fieldworkers screened the clients for eligibility using a brief pre-screening survey that captured a client’s age, the services he or she was accessing on the day, and the services that client had ever accessed at that facility, as well as interest in participating. Clients who were younger than 18 or were accessing or had ever accessed ART services for HIV treatment were excluded from the study, as per protocol-defined exclusion criteria.

Clients who met the inclusion criteria and were interested in participating in the survey were formally consented and asked to provide written informed consent. The informed consent process was administered by fieldworkers in either English, Zulu, Afrikaans, SeSotho or Venda, depending on each client’s preference, in a location that ensured visual and auditory privacy. During this process, the fieldworkers highlighted that the survey was a research study and was not a part of normal clinic services. They also emphasized that participation was voluntary and that participation or non-participation would not affect the services received at the clinic.

#### Collection of survey data

After written informed consent was obtained, client surveys (S2 Appendix) were conducted by the fieldworkers during waiting periods for services or after clinic visits in private locations within the facility, such as counselling rooms or vacant offices, to ensure privacy and confidentiality. Study data were collected and managed using REDCap electronic data capture tools hosted at the University of Witwatersrand [10]. REDCap (Research Electronic Data Capture) is a secure, web-based application designed to support data capture for research studies. Each survey was administered on the REDCap Mobile App by a trained fieldworker in the participant’s language of preference (English, Zulu, Afrikaans, SeSotho or Venda). The survey, which contained primarily closed- and a few open-ended questions, took approximately 30–45 minutes to complete. It covered participants’ demographics, sexual behaviours and HIV prevention practices, risk perception, oral PrEP knowledge, ever and current use of oral PrEP, perceptions and use practices, satisfaction with oral PrEP services (including wait times and interactions with providers), and beliefs about or experiences with side effects of oral PrEP. Survey participants were not reimbursed for their participation as reimbursement criteria was defined for time and travel costs, and since participants were recruited from the clinic and interviewed during waiting times, neither time or travel costs were incurred.

### Qualitative data collection

#### Recruitment

During the survey, all participants were asked if they would be interested in participating in an in-depth interview (IDI), and their answers were documented together with participant contact details. Approximately two months after survey completion, all participants who agreed to be contacted for an IDI were called by the private research firm to determine interest in participation and to set up a suitable time to conduct the interview. A maximum of five contact attempts were made for each participant. Three clients who did not participate in the survey did participate in an IDI. They were “walk-in” participants who visited the clinic and were recruited on the same day to participate in the qualitative phase of the study.

#### Collection of IDI data

IDIs were conducted one-on-one by five experienced female data collectors who were trained in research ethics, qualitative methods, interviewing skills, and the study protocol. Using a structured interview guide (S3 Appendix), data collectors explored the following topics with clients: oral PrEP decision-making, reasons for continuing or discontinuing oral PrEP, challenges with using PrEP, side effects, and perceptions of information, education, and communication materials (IEC). Initial survey findings were used to formulate specific questions to ask during the IDIs to lend detail and context to quantitative findings. Interviews were conducted in English, Zulu, SeSotho or Venda- at the clinic or another location determined by the participant. All participants provided written informed consent for participation in the IDI and permission to be audio-recorded, and all IDIs were audio recorded. Interviews ranged from 14 minutes to 64 minutes, with an average of 36 minutes. IDI participants were reimbursed R50 for their time and travel costs. Immediately following each interview, the data collector audio-recorded a brief summary of the interview and his or her reflections on it.

### Data management and analysis

Study survey data were managed using REDCap, and data were cleaned and analysed using Stata 13. Upon collection of all the surveys data from REDCap server was exported to STATA 13 and checked for missing or discrepant data. Out of 315 surveys, 16 were excluded due to missing data (more than 20% of questions unanswered) or discrepant data on oral PrEP status, leaving a sample size of 299 participants. Summary statistics and tabulations were computed for the entire sample and separately for participants who accessed services at MSM and SW sites.

Qualitative interviews were simultaneously translated to English and transcribed by professional transcriptionists. Four analysts developed a codebook of structural and emergent codes and compared coding until inter-coder agreement reached 80% [11,12]. The analysts coded the remaining transcripts in NVivo 11, checking in regularly regarding coding questions and updates to the codebook. The analysts used a standard memo template to display themes and supporting data, along with a summary of the results for each code or group of related codes [12]. A secondary analyst reviewed each memo to ensure accurate interpretation and representation of the data.

### Ethical considerations

Ethical approval from the Wits Human Research Ethics Committee (HREC) was received on 29 August 2017 (M160788). The protocol was simultaneously submitted to the Advarra (formerly Chesapeake) institutional review board (IRB) in fulfilment of Clinton Health Access Initiative (CHAI) organizational policy and was approved on 22 August 2017 (Pro00022378). The FHI 360 IRB signed an IRB Authorization Agreement authorizing the Chesapeake IRB to be the IRB of reference for this study. Facility managers at the selected facilities were presented with a letter from the NDoH indicating its support of the study and with the IRB approvals when the ACCESS investigators requested permission to conduct the study at those sites. Additionally, all study staff completed the NIH ethics certificate training.

## Results

### Study sample

Researchers surveyed 299 HIV-negative clients at the six SW and three MSM sites that had been implementing oral PrEP for a minimum of three months to a maximum of 16 months, with an average of 14 months for SW sites and 5 months for MSM sites. Based on self-identification, the sample comprised of 156 (52%) FSWs, 1 (0.3%) male sex worker (MSW), 80 (27%) MSM, 57 (19%) participants that identified as neither FSW, MSW nor MSM and 5 (2%) as Other (1 in sero-discordant relationship, 2 partners of sex workers, 1 former sex worker, 1 did not specify). The sample included 203 clients from SW sites and 96 clients from MSM sites (see Table 2). Out of the 299 clients enrolled, 260 had heard of PrEP and comprised 94 current users, 80 past users, and 86 never users. After completion of the survey, 275 clients were interested in participating in an IDI, 24% (n = 65) agreed to participate in interviews. Of the 76% (n = 210) who were not available, 48% did not answer the call, 19% had an invalid contact number, 7% were either working or too busy, and 2% subsequently refused. Of the 65 clients who agreed to participate, 59 interviews were scheduled, of which 29 were successfully conducted. Of the 29 clients interviewed, 20 were from SW sites, and 9 were from MSM sites. The primary reasons for interviews not being conducted with the remaining 30 clients who agreed to participate include working or being too busy (35.5%, n = 11), not answering the phone/voicemail (25.8%, n = 8), and being on holiday/visiting family (12.9%, n = 4).

**Table 2:** Survey sample characteristics.

	Participants from SW sites N = 203		Participants from MSM sites N = 96		Total N = 299	
	n	%	n	%	n	%
Age						
18–24	42	20.7%	23	24.0%	65	21.7%
25–34	114	56.2%	35	36.5%	149	49.8%
35+	47	23.2%	38	39.6%	85	28.4%
Gender						
Male	12	5.9%	88	91.7%	100	33.4%
Female	187	92.1%	4	4.2%	191	63.9%
Transgender man	1	0.5%	1	1.0%	2	0.7%
Transgender woman	3	1.5%	3	3.1%	6	2.0%
Education						
No schooling	5	2.5%	1	1.0%	6	2.0%
Up to primary	19	9.4%	3	3.1%	22	7.4%
Up to grade 11	110	54.2%	9	9.4%	119	39.8%
Matriculation^1^	61	30.0%	55	57.3%	116	38.8%
Tertiary	8	3.9%	28	29.2%	36	12.0%
Relationship status						
Single	155	76.4%	63	65.6%	218	72.9%
In a relationship/ Married	47	23.2%	23	24.0%	70	23.4%
Separated/divorced	1	0.5%	10	10.4%	11	3.7%

^1^Matriculation refers to completion of the 12th Grade, which marks completion of secondary school education

29 of the 299 clients were enrolled for an in-depth interview, of which 17 were current oral PrEP users, 3 were past users, and 9 had never used oral PrEP.

### Participant socio-demographics

The majority of survey participants were 25 years and older. Approximately two-thirds (63.9%) were female, and one-third were male (33.4%) (See Table 2). The sample was primarily female (92.1%) at SW sites and primary male (91.7%) at MSM sites. Level of education varied by site, with 30% of participants at SW sites having completed the 12^th^ grade of schooling (graduated from high school) compared to 57.3% of participants at MSM sites. Participants at MSM sites also displayed higher levels of tertiary education (29.2%) compared to participants at SW sites (3.9%). The majority (72.9%) reported their relationship status as single.

Of the 29 clients interviewed in IDIs, 11 were male and 18 were female.

### Perceived HIV risk and oral PrEP uptake

The perceived risk of being infected with HIV was higher among participants at SW sites (61.6%) compared to MSM sites (35.4%). SWs (80.3%) were more likely to be exchanging sex for money than MSM (12.5%), a known HIV risk factor. Most participants in the sample (97.7%) had been tested for HIV, with over half (55.5%) reporting being tested in the past three months (see Table 3). Transactional sex was reported by 80.3% of participants at SW sites and 12.5% of participants at MSM sites.

**Table 3:** Risk perception and oral prep uptake.

	SW sites N = 203		MSM sites N = 96		Total N = 299	
	n	%	n	%	n	%
Ever had an HIV test						
Yes	198	97.5%	94	97.9%	292	97.7%
No	5	2.5%	2	2.1%	7	2.3%
Last HIV test	N = 198		N = 94		N = 292	
Less than 3 months ago	121	61.1%	41	43.6%	162	55.5%
3–6 months ago	51	25.8%	41	43.6%	92	31.5%
7–11 months ago	14	7.1%	5	5.3%	19	6.5%
12 months ago	8	4.0%	3	3.2%	11	3.8%
More than 12 months ago	4	2.0%	4	4.3%	8	2.7%
Do you believe you are at risk of HIV?						
Yes	125	61.6%	34	35.4%	159	53.2%
No	66	32.5%	41	42.7%	107	35.8%
Not sure	12	5.9%	21	21.9%	33	11.0%
Exchanged sex for money, goods, or services in the past year						
Yes	163	80.3%	12	12.5%	175	58.5%
No	39	19.2%	84	87.5%	123	41.1%
Previously heard of PrEP						
Yes	175	86.2%	85	88.5%	260	87.0%
No	28	13.8%	11	11.5%	39	13.0%
Oral PrEP use among those who had heard of PrEP	n = 175		n = 85		n = 260	
Current user	66	37.7%	28	32.9%	94	36.2%
Past user	50	28.6%	30	35.3%	80	30.8%
Never user	59	33.7%	27	31.8%	86	33.1%

The majority (87%) of participants had heard of PrEP. Of these slightly over a third (36.2%,) were currently using oral PrEP, slightly under a third (30.8%) had used oral PrEP but had stopped, and a third (33.1%) had never used oral PrEP.

### Factors influencing lack of uptake of PrEP among never users

#### Survey

Among participants who had never used oral PrEP, the primary reason reported for lack of uptake was not being offered oral PrEP by a healthcare provider (57%, n = 49). Of those who were not offered oral PrEP, over half (56%, n = 27) perceived themselves to be at risk of HIV. The remaining 43% (n = 37) of participants had been offered oral PrEP but had declined. The primary reason for declining to use oral PrEP was fear of side effects (41.7%, n = 15) (see Table 4).

**Table 4:** Reasons for lack of uptake of oral PrEP among never users.

	SW sites		MSM sites		Total	
	n	%	N	%	n	%
Reason for not initiating oral PrEP	N = 60		N = 26		N = 86	
Never been offered	28	46.6%	21	80.8%	49	57.0%
Declined	32	53.3%	5	19.2%	37	43.0%
Reason for declining oral PrEP (select one)*	N = 31		N = 5		N = 36	
Afraid of side effects	13	41.9%	2	-	15	41.7%
Afraid of stigma	4	12.9%	0	-	4	11.1%
Concerns with taking pills/ability to adhere	4	12.9%	0	-	4	11.1%
Only have one faithful sexual partner	2	6.5%	1	-	3	8.3%
Clinic is too far	3	9.7%	0	-	3	8.3%
My family did not want me to use it	1	3.2%	0	-	1	2.8%
Other	3	9.7%	2	-	5	13.9%

*One SW site participant did not answer this question. Responses from the MSM sites are displayed as absolute numbers due to the small sample size.

#### In-depth interviews

All the never users knew about oral PrEP, and most of them had received counseling on PrEP. Those who had been counseled had various reasons for not initiating immediately, such as not having time that day or wanting additional information from a friend who was using PrEP. Five of the nine never users indicated that they were interested in using oral PrEP in the future.

A few never users were concerned about potential side effects. Some of them seemed open to the possibility of taking oral PrEP, especially if they did not experience side effects or if it would be possible to take oral PrEP less than daily. Only two clients—both from MSM sites—had decided not to use oral PrEP because of their concerns about side effects and daily pill taking. They had witnessed or experienced negative side effects from post-exposure prophylaxis (PEP) and antiretroviral therapy.

*[Oral PrEP is] a good thing but … you forget sometimes to take it*. *It’s a good thing*, *but it’s too much of work*.

Never user-1, MSM site, 26-year-old male

*I think I won’t take [oral PrEP]*, *because my older brother is taking ARVs and […] after taking the pill*, *he eats too much […] which I don’t like*. *Sometimes he complains about being weak*, *he’s weak and he’s not feeling well*. *Even though he looks healthy*, *he’ll have a few complaints about his body*. *I think it’s because of the ARVs*. *Everything has negative effects*, *especially ARVs*. *That’s what I hate about ARVs and those are so many reasons to hate PrEP*.

- Never user-2 MSM site, 21-year-old male

### Factors influencing initiation and continuation of oral PrEP

#### Survey

The primary reasons for initiating oral PrEP were all related to perceived risk associated with sexual activity, which were reinforced by similar reasons for continuing PrEP (see Table 5). The majority of participants (87.9%, n = 153) also noted that printed IEC materials influenced their decision to initiate PrEP. Moreover, the majority (93.1%, n = 81) stated that printed materials helped them continue using PrEP. The NDoH PrEP fact sheet was cited as the most popular format of printed material that influenced decision making.

**Table 5:** Reasons for initiating and continuing oral PrEP.

	SW sites		MSM sites		Total	
	n	%	n	%	n	%
Reason for starting oral PrEP (select one)*	N = 113		N = 18		N = 131	
I am sexually active	39	34.5%	10	-	49	28.2%
I feel that I am at risk for HIV	29	25.7%	4	-	33	19.0%
I have multiple sexual partners	27	23.9%	4	-	31	17.8%
I have clients who I believe could be HIV+	10	8.8%	0	-	10	5.7%
I have clients who do not want to use condoms	4	3.5%	0	-	4	2.3%
Other	4	3.5%	0	-	4	2.3%
Reason to continue oral PrEP (select multiple)**	N = 66		N = 27		N = 94	
I am sexually active	34	51.5%	24	88.9%	58	61.7%
I feel that I am at risk for HIV	34	51.5%	11	40.7%	45	47.9%
I have multiple sexual partners	32	48.5%	6	22.2%	38	40.4%
I have clients who I believe could be HIV+	23	34.8%	2	7.4%	25	26.6%
I have clients who do not want to use condoms	8	12.1%	0	0.0%	8	8.5%
I haven't had any problems	3	4.5%	0	0.0%	3	3.2%
Other	4	6.1%	1	3.7%	5	5.3%
Printed information that influenced or helped with decision to use PrEP? (select multiple) ***	n = 114		n = 58		n = 172	
Posters	27	23.7%	14	24.1%	41	23.8%
Fact sheet	34	29.8%	16	27.6%	50	29.1%
FAQs	22	19.3%	2	3.4%	24	14.0%
PrEP initiation packet	19	16.7%	2	3.4%	21	12.2%
Pocket book	26	22.8%	6	10.3%	32	18.6%
Did not influence my decision	7	6.1%	19	32.8%	26	15.1%
Other	1	0.9%	0	0.0%	1	0.6%
Printed information that helped you to continue with use of PrEP? (select multiple) ****	n = 61		n = 25		n = 86	
Posters	13	21.3%	9	36.0%	22	25.6%
Fact sheet	23	37.7%	14	56.0%	37	43.0%
FAQs	19	31.1%	2	8.0%	21	24.4%
PrEP initiation packet	13	21.3%	6	24.0%	19	22.1%
Pocket book	17	27.9%	7	28.0%	24	27.9%
Did not help me to continue with my use of PrEP	4	6.6%	6	24.0%	10	11.6%

* Three responses were invalid for SW, 27 responses were invalid for MSM, and 13 were missing. Responses considered to be invalid included those under the “other” selection response. These responses indicated a misunderstanding of the question.

** One MSM site participant did not answer this question.

***This question was asked on the Zulu form as a select one, and on the English form as a select multiple. 2 responses were missing for SW site participants

****Five FSW site participants and 3 MSM site participants did not answer this question.

#### In-depth interviews

As in the survey, the main reason that participants—including current, past, and never users—described using or intending to use oral PrEP was that the majority perceived themselves to be at risk of HIV and wanted to remain HIV-negative. Several wanted additional protection from HIV beyond only using condoms because they had experienced challenges such as partners refusing to use condoms or condoms bursting. Many also described sexual behaviors or relationships that put them at risk, including having partners with unknown HIV status or partners they did not trust, having multiple partners or open relationships, involvement in sex work, or having a partner living with HIV. Participants often described initiating oral PrEP for a variety of reasons that were interlinked. For example, one current user involved in sex work described her desire to have additional protection from HIV if a condom failed or a client forced her to have sex:

*I was not using anything*. *Then*, *the other sister of mine*, *she explained me*, *“you see this job is risking*. *The condom gonna burst*, *and some people they’re gonna force you [to have sex]*. *We don’t know*. *So*, *it’s better for you to prevent*, *because when the condoms burst*, *we are not taking PrEP*, *it’s risky*. *So*, *it’s better to take PrEP*. *When the condom burst*, *or something happen*, *you know that you are on the safe side…*

- Current user-1 SW site, 23-year-old female

Another current user described the desire for protection in a monogamous relationship:

*Currently I’m in a monogamous relationship […] I’ve always be very safe person*. *[But] I can’t control my partner […] so I would rather protect myself*.

- Current user-1 MSM site, 31 year-old male

Many participants described how they received information or encouragement from others that influenced their decisions to use oral PrEP or their intentions to use it in the future. Notably, all but two of these participants were from SW facilities. Many participants at SW facilities described talking to a provider who gave them comprehensive information, helped them understand PrEP, and encouraged them to use it. Some stated that a provider played “a huge role” in their decisions to use PrEP:

*I can say that firstly*, *I didn’t care about PrEP*. *They had said if I wanted to*, *then I should let them know if my mind had changed about taking PrEP*. *And [they] would call me to encourage me about PrEP*. *I ended up taking it*. *The way they explained to me on the first day made me wish to explain to my friends the way she had explained it*. *[…] [Provider’s name] explained to me very well*. *Even what I asked her*, *she would explain to me and asked me if I had understood*. *If I said I did*, *she would ask me what’s she had said so that she will make sure that I heard*.

- Current user-2 SW site, 19-year-old female

Some participants also received information or encouragement from friends, sisters (SW only), a partner (MSM only), and peers (MSM only). Some of these individuals were oral PrEP users, who shared their experiences using it and answered questions. One participant described being unsure about oral PrEP after receiving counseling at the clinic, but talking to her friend helped her decide to use PrEP:

*My ex-boyfriend was the one who was on PrEP and he suggested that why don’t you also try it*. *I had heard about it but I was kind of skeptical about taking pills every day and the tediousness of it*, *but I did some research on it and … I went to the clinic and then I started the programme three months ago*.”

- Current user-2 MSM site, 29 year old male

*I couldn’t take it after they taught us*, *because I wanted to think about it*, *to understand better what kind of pill it was*. *[…] I wanted to know better about it*. *You hear people talking about prevention*, *and I didn’t know if it was just a Panadol [acetaminophen] or what*. *Then I asked one friend to show me what she uses for HIV [prevention]*. *She removed a big bottle and showed me blue pills inside*. *[…] I told her I wanted it and she told me I could get it at [clinic name]*

- Never user-1 SW site, 25-year-old female

For some current users, informational materials seemed to play an integral role in their decision to use PrEP. For example, one participant said that seeing the materials piqued his interest in oral PrEP and helped him decide to go to the clinic to get more information. Another participant said that after initiating oral PrEP, she found the posters really motivating because they normalized oral PrEP use:

*I will say the full truth*. *You see PrEP*, *I didn’t care about it*. *But when I had joined it and seeing those posters*, *I saw that it was something real*, *it’s something that a lot of people use*. *I became encourage with that*.

- Current user-2 SW site, 19-year-old female

Other current users found the materials informative and studied them in detail when making the decision to initiate oral PrEP. One client took the materials home after her initial clinic visit, studied them, and then returned to ask the provider detailed questions to be sure she had a thorough understanding before initiating oral PrEP.

During IDIs some current, past, and never users provided suggestions for improving existing IEC materials and developing additional materials. They indicated a need for materials addressing side effects and their management, the translation of all IEC materials into the 11 official languages of South Africa and making the materials available online and through social media channels.

Many current users described a sense of motivation and determination to protect their health and remain HIV-negative as their main reasons for continuing to use oral PrEP. Participants talked about wanting to “protect myself,” “stay healthy,” “keep my status,” and “cover the bases.” Some were motivated by fear or worry, knowing that discontinuing oral PrEP would “put my life at risk” or they “might be infected.”

…*these pills are good for me*. *I would still like to continue using them because I don’t want to contract HIV*. *I would like to continue protecting myself*.

- Current user-3 SW site, 19-year-old female

Some described behaviors and relationships that put them at risk of HIV and motivated them to continue using oral PrEP, including having multiple partners, knowing a partner is HIV-positive, not knowing a partner’s HIV status, not trusting a partner, and forgetting to use condoms.

*It’s because I don’t trust my partner*. *I don’t know who else he sleeps with*, *and that’s why I drink my pills and continue using them at all times*.

- Current user-3 SW site, 19-year-old female

*We did use protection and stuff*, *but I’m pretty sure with the open relationship thing*, *there might have been occasions where I did actually have intercourse with somebody that was also HIV positive and didn’t tell me about it*. *So*, *the protecting yourself component as an additional protection is […] probably the main factor [for continuing PrEP]*, *actually*.

- Current user-1 MSM site, 39 year old male

### Factors influencing discontinuation of PrEP

#### Survey

The majority (73.8%, n = 59) of participants who discontinued oral PrEP cited side effects as the primary reason for discontinuation, followed by feeling stigmatized (18.8%, n = 15) (see Table 6). Compared to current users, more past users reported experiencing side effects (95% versus 59%). Only 15% of past users stated that the side effects were tolerable, and 83% said that the side effects they had experienced had affected daily life as per S1 Fig. The most commonly reported side effects that were considered intolerable by past users (n = 47) were stomach pain (89.4%, n = 42), vomiting (42.6%, n = 20), and nausea (40.4%, n = 19). Discontinuation due to side effects happened on average within the first five months of use.

**Table 6:** Reasons for discontinuing PrEP.

	SW sites		MSM sites		Total	
	n	%	n	%	n	%
Reason to discontinue PrEP (select multiple)*	N = 50		N = 30		N = 80	
Side effects were too much	33	66.0%	26	86.7%	59	73.8%
I felt stigmatized	12	24.0%	3	10.0%	15	18.8%
Challenges with accessing PrEP	6	12.0%	2	6.7%	8	10.0%
Concerns with taking pills/ability to adhere	1	2.0%	2	6.7%	3	3.8%
Only have one faithful sexual partner	0	0.0%	2	6.7%	2	2.5%
My partner told me to stop using PrEP	1	2.0%	1	3.3%	2	2.5%
Became pregnant	2	4.0%	0	0.0%	2	2.5%
Other	3	6.0%	0	0.0%	3	3.8%

*Eight SW site participants and one MSM site participant did not answer this question.

#### In-depth interviews

Both current and past users employed a variety of strategies to deal with side effects. A few said that they went to the clinic or contacted a provider via phone or WhatsApp. Providers advised them to continue taking oral PrEP and said that the side effects would wear off eventually or suggested changing the timing of daily pill taking. Some participants started taking the pills at night instead of in the morning, or they took PrEP after meals. A few participants said that they did not go to the clinic when they experienced side effects because they had been informed about side effects by the provider or IEC materials:

*“They explained to me when I started that you might have side effects*, *and there are pamphlets there that explains side effects*, *so I wasn’t struggling that much”*

- Current user-2 MSM site, 29-year-old male

A couple of participants mentioned stopping or taking a break from oral PrEP because of the side effects they were experiencing. Interestingly, a few participants talked about how people taking oral PrEP have different experiences with side effects, either because some have worse side effects than others or because they have a different mindset about dealing with them. A current user from a SW site said that oral PrEP is like contraceptives, because people experience different side effects from the same contraceptive. Another current user thought that people make different decisions about how they react to side effects:

*“You know*, *some people in life*, *we are different*. *And we think different*. *[…] I just say […] I’m continuing taking this*, *until I stop this thing I’m doing it”*

-Current user-1 SW site, 23-year-old female

Current, past, and never users talked about two types of stigma associated with oral PrEP—one related to the assumption that people taking oral PrEP are “promiscuous” or have multiple partners, and another related to misperceptions that people who are on oral PrEP are HIV positive and are taking ART. Only two participants talked about their own personal experience with PrEP-related stigma: one MSM respondent said he broke up with his partner, who believed that he was HIV-positive because he was taking oral PrEP. He also had disagreements with his father, who thought taking oral PrEP meant he was “playing around.” A past user said that people were “*spreading a lot of rumors about what pills we were collecting from that vehicle*” (Past user-1 SW site, 24-year-old female). Past and never users did not directly link the experienced or anticipated stigma with discontinuation or lack of uptake.

Only three past users, all from SW facilities, participated in IDIs. They each had a different reason for discontinuing oral PrEP. One became pregnant and was counseled to discontinue oral PrEP, per national guidelines, until after she gave birth and the baby reached a certain stage of growth. Another participant discontinued after leaving sex work because she found a partner who was supporting her, and therefore her perceived HIV risk was reduced. Both women intended to use oral PrEP again. The third past user said she discontinued because she was counseled that if she missed any doses, she would have to reinitiate, which she found too burdensome. She said she would use oral PrEP again in the future if she could miss a dose without reinitiation or if it were available as an injection:

*I am someone who liked partying*, *and on some day*, *would forget to take my pills*, *and they told us not to stop taking it for 28 days and if you skipped a day*, *when you go back to taking them*, *you would start back at one*, *as if you had never [been] taking it before*.

-Past user-1 SW site, 24-year-old female

All three past users experienced side effects from oral PrEP, but they did not discontinue because of the side effects.

### Combination prevention amongst current and past PrEP users

#### Survey (see Table 7)

**Table 7:** Condom use at last sex at SW sites by PrEP use status.

	Current PrEP Users		Past PrEP Users		Never Used PrEP		Total	
*Condom Use at Last Sex*	n	%	n	%	n	%	n	%
Main Partner	N = 37		N = 28		N = 29		N = 94	
Yes	15	40.5%	6	21.4%	15	51.7%	36	38.3%
No	20	54.1%	21	75.0%	13	44.8%	54	57.4%
I don’t remember	2	5.4%	1	3.6%	1	3.4%	4	4.3%
Casual Partner	N = 25		N = 21		N = 21		N = 67	
Yes	18	72.0%	12	57.1%	17	81.0%	47	70.1%
No	6	24.0%	7	33.3%	4	19.0%	17	25.4%
I don’t remember	1	4.0%	2	9.5%	0	0.0%	3	4.5%
Sex Worker Client	N = 56		N = 43		N = 49		N = 148	
Yes	45	80.4%	32	74.4%	36	73.5%	113	76.4%
No	7	12.5%	5	11.6%	7	14.3%	19	12.8%
No response	4	7.1%	6	14.0%	6	12.2%	16	10.8%

In surveys at SW sites, over 75% of participants said that they used a condom the last time they had sex with a client; these proportions were similar among current (80.4%), past (74.4%), and never (73.5%) users. Among those with main (n = 94) or casual (n = 67) partners, condom use was higher with casual partners overall, and was higher for never (81% casual/51.7% main) and current users (72% casual/40.5% main) compared to past users (57.1% casual/21.4% main). Condom use was lowest with main partners, and in IDIs some participants described that in steady relationships it was challenging to use condoms. Most current users felt it was easy to use PrEP and condoms simultaneously with main partners (90%) and clients (95%). However, in IDIs many noted that clients removed condoms and offered more money to "trick" or "tempt" participants into having sex without them, which could explain why, when asked which method worked better for them (condoms, PrEP or both), 70% of current users preferred both methods. However only 19% of past users preferred both, and 72% preferred condoms alone.

## Discussion

Recent studies and programmatic data from many countries implementing oral PrEP have highlighted challenges in promoting uptake and continuation. In Kenya, for instance, 44% of eligible SWs and 33% of eligible MSM initiated oral PrEP [13]. In South Africa, overall uptake represents 16% of those who are offered PrEP [7]. In San Francisco in the United States, uptake of oral PrEP has been 40% among MSM and 15% among transgender women, alongside very high awareness of oral PrEP (97% of MSM and 79% of transgender women) [14]. Moreover, significant issues with oral PrEP continuation have been identified, with 50% or more of clients discontinuing within the first one to six months of use at sites in Kenya, South Africa, and the United States [15, 16]. Similar rates of continuation were also seen among clients who participated in the ACCESS study.

Our results suggest that never users may not start PrEP because they are not being offered oral PrEP. This finding points to missed opportunities to provide counselling and offer oral PrEP to clients who perceived themselves to be at risk of HIV. Providers may need support and additional training to help them identify opportunities to dialogue with clients about their risk of HIV, encourage testing, and discuss potential options for HIV prevention, such as PrEP.

Consistent with other evidence [13,17,18], these results suggest that understanding risk associated with sexual activity is the first step to oral PrEP initiation and a motivator for continuing on the method. Clients in this study associated risk due to sexual activity with being involved in sex work, having multiple partners, and engaging with partners who could be HIV-positive. Finding ways to empower more people to perceive their own risk more accurately—even as it may be changing frequently—is critical for sustained HIV prevention. Risk assessment tools are being utilized in some programs [19]. However, some believe that risk perception may not be a message that resonates and that providers should focus on messaging about sexual health and other reasons why people may want to take PrEP, such as to reduce anxiety, enhance intimacy, or promote a better future [20]. Further research on perceptions and messaging for both women and men is currently underway in South Africa such as the PSI Test and Treat Initiative for Men and the Prevention Market Manager AGYW studies [21].

Among other motivators for clients to initiate and continue PrEP, IEC materials played an important role in clients’ decision-making. The ACCESS study results suggest that well-designed, empowering, and concise IEC materials that have been developed with potential user input can be influential in improving client knowledge and encouraging continued use of PrEP. The finding that 13% of clients interviewed in sites offering oral PrEP had never heard of this prevention option, however, suggests more attention is still needed for PrEP promotion. In response to participants’ suggestions, IEC materials are being developed to address side effects and their management, stigma, cycling on and off oral PrEP, and information about and support for adherence. A website, www.myprep.co.za, has been developed and messaging about oral PrEP is being disseminated through various social media channels.

Side effects seem to play a role in lack of uptake and discontinuation of PrEP. With regards to uptake, side effects were cited as a secondary reason for lack of initiation, following not being offered PrEP. Additionally, our results suggest that side effects were the primary reason why past users had stopped using oral PrEP within the first five months of use. Side effects have been reported as a barrier to uptake and continuation in other studies [15, 16], but they were more of a concern in this study. Users in this sample who discontinued oral PrEP found side effects challenging, with many citing the side effects as intolerable and affecting daily life. Clients who continued using oral PrEP appeared to tolerate side effects differently from those who stopped using it. They also sought help from the clinics and employed methods to cope with side effects, such as changing the time of day when they took their pills. This finding highlights the importance of support to effectively manage and overcome side effects. Client recall of counselling on management of side effects suggests that this topic was not well covered: 41% of clients remembered discussing side effects management with counsellors. This finding reveals the critical need to train and support health providers, as well as users and potential users, not only about the side effects but also on their management. Provider training in oral PrEP provision has been adapted in South Africa to include further training on how to counsel and manage clients who return to a facility with side effects, and as noted above IEC materials have been developed with more information on side effects.

Smith *et al* (2012) conducted a study in the United States among young adults using oral PrEP, and found concerns over ARV-based stigma and burden of daily pill taking [22]. In the ACCESS study, similar concerns were cited by a small number of survey participants as reasons for not initiating or continuing oral PrEP use. A small number of in-depth interview participants also mentioned these concerns but did not cite them as reasons for lack of uptake or continuation. These issues are worth monitoring in future studies and in oral PrEP programs.

Clients who had discontinued PrEP reported lower levels of condom use at last sex with main or casual partners compared to current and never users of PrEP. This finding suggests that this group may struggle with aspects of adherence, such as the consistent use of condoms or continued use of a daily regimen, and may need additional support to sustain oral PrEP use or other prevention during periods of risk. It also suggests that past users of oral PrEP may be a group to target for future HIV prevention interventions currently under review that are less user-dependent, such as the dapivirine ring for women or oral PrEP on demand for MSM.

## Conclusion

This study provides valuable insights on early rollout of PrEP of how clients perceive oral PrEP and where to target efforts to improve the uptake of this highly effective HIV prevention product. By identifying strengths and areas for improvement, the ACCESS study has generated evidence that can be used to guide high quality scale-up in South Africa and may be instructive for other countries’ efforts to expand quality access to oral PrEP.

## Limitations

The ACCESS study findings could be influenced by the fact that the study was conducted during the early phase of national PrEP implementation, at a time of limited uptake and client reservations about PrEP. The cross-sectional study design which did not allow for observations of condom use before and after PrEP initiation, as well as aspects around cycling on and off PrEP and discontinuing over a period of time. Also, the study focused on SW and MSM sites and was based on a convenience sample of clients within each facility, and therefore may not be generalizable to all oral PrEP implementing sites. Also, convenience sampling may have introduced sampling bias, as clients present in the clinic may have distinctly different attitudes or perceptions towards PrEP. Additionally, given that more SW sites were implementing PrEP compared to MSM sites, the sample was equal and quantitive data was not available for the three “walk-up” participants recruited on site for the qualitative interviews. Since the surveys were administered by interviewers, responses from participants could be subject to social desirability bias given the sentence nature of the questions. We however aimed to minimize this through training of our interviewers on rapport building. It would be valuable to conduct a similar study in sites providing comprehensive sexual and reproductive health services to AGYW.

For the IDIs, we were unable to recruit enough past users to reach thematic saturation in this participant group, and all the past users who participated in IDIs were SWs. Additional IDIs with people who have discontinued oral PrEP could provide a deeper understanding of the main reasons for discontinuation indicated in our survey. Nonetheless, the experiences past users described during the IDIs—getting pregnant, leaving sex work, and confusion over effective use instructions do provide some insight. In additional, we were not able to reach thematic saturation for all themes, likely because we had a heterogeneous study population that had diverse experiences (current/past/never users and SW/MSM sites).

## Supporting information

S1 Fig Tolerability of side effects amongst current and past users. (JPG)

S1 Appendix COREQ checklist. (PDF)

S2 Appendix Survey data collection tool. (PDF)

S3 Appendix In-depth interview guides. (PDF)
