# Supplementary figures and images for: Factors influencing uptake, continuation, and discontinuation of oral PrEP among clients at sex worker and MSM facilities in South Africa
Source: PLoS One. 2020 Apr 30;15(4):e0228620. doi: 10.1371/journal.pone.0228620 (PMC7192496; doi:10.1371/journal.pone.0228620)

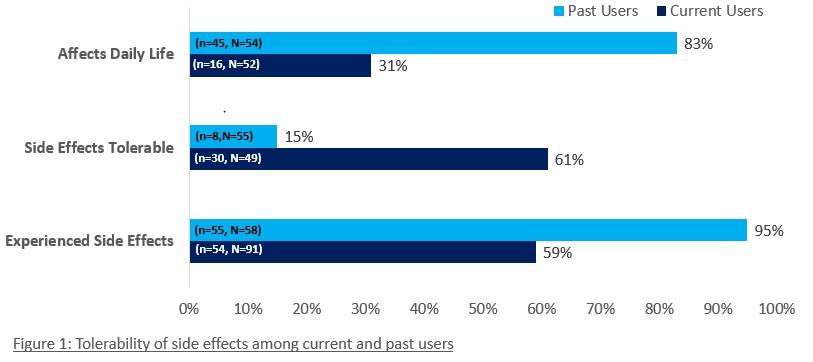

Supplement: S1 Fig — (JPG) [file pone.0228620.s001.jpg]
