# Supplementary material for: Factors influencing uptake, continuation, and discontinuation of oral PrEP among clients at sex worker and MSM facilities in South Africa
Source: PLoS One. 2020 Apr 30;15(4):e0228620. doi: 10.1371/journal.pone.0228620 (PMC7192496; doi:10.1371/journal.pone.0228620)
Supplement: S2 Appendix — (PDF) [file pone.0228620.s003.pdf]

Prescreening Survey Access

Record ID

____

Facility name

- ◯ NSA Hoedspruit/Limpopo
- ◯ NSA Musina /Limpopo
- ◯ PHRU/Soweto
- ◯ THCA eThekwini/Kwazulu Natal
- ◯ THCA uMkhanyakude/kwaZulu Natal
- ◯ WRHI Tshwane/ Guateng
- ◯ ANOVA Health4Men Yeovil/ Guateng
- ◯ ANOVA Health4Men Woodstock/Western Cape
- ◯ OUT Ten 81/Gauteng

Participant ID#

____
(Put the right code)

INTRODUCTION:
My name is [insert name]. I am conducting a service evaluation at this facility. Would you mind if I ask you a few questions?

Date of interview

____

Date of birth

____

Age of participant

____

"THIS PARTICIPANT IS YOUNGER THAN 18 AND INELIGIBLE" PLEASE DO NOT PROCEED!!!

What services are you accessing today?

- ☐ Family Planning
- ☐ PrEP
- ☐ ARVs
- ☐ STI's
- ☐ Other

(select all that apply)

"THIS PARTICIPANT IS INELIGIBLE". DO NOT PROCEED!!!

If service is other, please specify

____

What services have you ever accessed at this facility?

- ☐ Family Planning
- ☐ PrEP
- ☐ Arv's
- ☐ STI's
- ☐ Other

(Mark all that apply)

If other, please specify

____

Are you currently using PrEP?

◯ Yes
◯ No

INVITATION TO PARTICIPATE:
We are conducting a survey on HIV prevention services. We would like to know your views regarding HIV prevention and services received.

Would you like to participate in the survey?

◯ Yes
◯ No

Participant Demographics

Record ID ______________________

PARTICIPANTS' DEMOGRAPHICS
We will begin this survey by asking you questions about yourself.

Date of Interview ______________________

How do you identify your gender?
- ◯ Male
- ◯ Female
- ◯ Transgender man
- ◯ Transgender woman
- ◯ Other

(How do you identify yourself?)

Other, specify ______________________

What is your nationality?
- ◯ South African
- ◯ Non-South Africa

Please specify country ______________________

What is your ethnicity?
- ◯ Zulu
- ◯ Xhosa
- ◯ Ndebele
- ◯ Swati
- ◯ Sotho
- ◯ Pedi
- ◯ Tswana
- ◯ Venda
- ◯ Tsonga
- ◯ Other

If other, please specify? ______________________

What is your relationship status?
- ◯ Single/Never Married
- ◯ Casual partnership/s
- ◯ In a relationship/Not cohabiting
- ◯ In a relationship/Cohabiting
- ◯ Married/Cohabiting
- ◯ Divorced/Widowed
- ◯ Separated

What is your highest level of education completed?	◯ No Schooling<br>◯ Up to Primary<br>◯ Up to grade 11<br>◯ Matric<br>◯ Tertiary<br>(Interviewer to ask participant before moving to the next section: Are you happy to continue with the survey, do you have any questions at this time?)

Hiv Risk And Sexual Behaviour

Record ID ______________________

HIV RISK PERCEPTION AND HIV PREVENTION
We would now like to ask you some questions about what you think of HIV and if you think you are at risk. We also want to find out what you think about HIV prevention.

***DO NOT PROMPT RESPONSES!!**

What are the different ways which one can prevent HIV infection?

- ☐ HIV cannot be prevented
- ☐ Abstaining from sex
- ☐ Being faithful to one sexual partner
- ☐ Using condoms consistently
- ☐ Using gloves to avoid contact with blood
- ☐ Male circumcision
- ☐ Microbicides
- ☐ Oral PrEP
- ☐ PEP
- ☐ Not sharing needles
- ☐ I don't know
- ☐ Other specify

(Mark all that apply)

Please specify other ______________________

Do you think you are at risk of getting HIV?

- ◯ Yes
- ◯ No
- ◯ Not sure

Why do you rate your risk in this way?

- ◯ I abstain from sex
- ◯ I always use a condom
- ◯ I only have one sexual partner
- ◯ I trust my sexual partner
- ◯ I don't always use a condom
- ◯ I have more than one sexual partner
- ◯ I don't trust my sexual partner
- ◯ I don't know
- ◯ Other

Other ______________________

Have you ever tested for HIV?

- ◯ Yes
- ◯ No
- ◯ I don't know

When last did you test for HIV?	◯ Less than 3 months ago<br>◯ 3-6 months ago<br>◯ 7-11 months ago<br>◯ A year ago<br>◯ More than a year ago<br>(Interviewer to ask participant before moving to the next section: Are you happy to continue with the survey, do you have any questions at this time?)

SEXUAL BEHAVIORS AND HIV PREVENTION PRACTICES

We would like to ask you about your sexual partner/s and how you prevent HIV with your partner/s. This section refers to partners outside of sex work related sexual partners.

Sexual behaviors and Prevention Practices with Partners (Social)

***DO NOT PROMPT RESPONSES!!**

In the past year, have you exchanged sex for money, goods or service?	◯ Yes<br>◯ No
Do you identify as:	◯ Female Sex Worker<br>◯ Male Sex Worker<br>◯ Men who have sex with men [MSM]<br>◯ None of the above<br>◯ Other
Other, specify	__________________________________
How many sexual partners (not related to sex work) do you currently have?	◯ None (Sex Worker)<br>◯ None (Not Sex Worker)<br>◯ One (Sex worker)<br>◯ One (Non- sex worker<br>◯ two (Sex worker)<br>◯ two (Non- Sex worker)<br>◯ More than 2 (Sex worker) (specify how many?)<br>◯ More than 2 (Non- sex worker)
More than 2, please specify	__________________________________
More than 2 non-sex worker, please specify	____
Do you have a main partner (non-paying/emotional partner?)	◯ Yes<br>◯ No
Thinking of the last time you had sex with your main partner, did you use a condom?	◯ Yes<br>◯ No<br>◯ I don't remember
Do you have a casual partner or partners (non-paying/emotional partner)?	◯ Yes<br>◯ No (Sex Worker)<br>◯ No (Not Sex Worker)

Thinking of the last time you had sex with a casual partner, did you use a condom?	◯ Yes<br>◯ No<br>◯ I don't remember

Sexual Behaviors and Prevention Practices with Partners (For Sex Workers)

If identified as female or male sex worker, we would like to ask you some questions about your work and how to practice HIV prevention with your clients

***DO NOT PROMPT RESPONSES!!**

For how long have you been doing sex work?	◯ Less than 6 months<br>◯ 7 months to a year<br>◯ 1-2 years<br>◯ More than 2 years
Where do you most often work from?	◯ From my home<br>◯ from client's home<br>◯ From a bar/tavern/brothel<br>◯ Wherever convenient for client<br>◯ Other
Other, specify	____________________________<br>(please write location)
How many clients do you normally see in a day on average?<br>(Note to Interviewer: do not read out response options.)	◯ 1<br>◯ 2<br>◯ 3<br>◯ More than 3 (specify)<br>(Write the number of other in the next question)
More than 3, please specify number	____________________________
Thinking of the last time you had sex with a client, did you use a condom?	◯ Yes<br>◯ No

Sexual Behaviors and Prevention Practices with Partners and Clients (Experience with violence) (For all survey participants)

***DO NOT PROMPT RESPONSES!!**

Within the last year, have you experienced any violence from one of your sexual partners (not sex work partner)? This could include verbal, emotional, physical, economic or sexual violence.	◯ Yes (sex worker)<br>◯ Yes (not sex worker<br>◯ No (sex worker)<br>◯ No (not sex worker)
What type of violence did you experience from your sexual partner/s (not sex work partner)?	☐ Emotional/Verbal<br>☐ Physical<br>☐ Economic<br>☐ Sexual<br>☐ Other<br>(Mark all that apply)

Other, please specify	__________________________________
Within the last year, have you experienced any violence during sex work? This could include verbal, emotional, physical, economic or sexual violence.	◯ Yes<br>◯ No
What type of violence did you experience during sex work?	☐ Emotional<br>☐ Physical<br>☐ Economic<br>☐ Sexual<br>☐ Other<br>(Mark all that apply)
Other, please specify	__________________________________<br>(Interviewer to ask participant before moving to the next section: Are you happy to continue with the survey, do you have any questions at this time?)

Oral Prep Knowledge

Record ID ______________________________

ORAL PREP KNOWLEDGE, PERCEPTIONS AND BELIEFS (For everyone)

We would like to ask you some questions about what you know about oral PrEP

Knowledge of Oral PrEP

***DO NOT PROMPT RESPONSES!!**

Oral PrEP is a medication that a person takes daily to prevent becoming infected with HIV. Have you heard of this medication?

◯ Yes
◯ No
(If answer is No end survey and refer to service provider)

(Note to Interviewer: If response is NO, Ask if respondent has any questions Refer to peer educator or healthcare provider if participant is interested in finding out more.)

Where did you first hear about oral PrEP from?

☐ Clinic
☐ Friends/Family members
☐ Colleagues
☐ Social media
☐ Internet
☐ Radio/TV/Newspaper
☐ Printed materials (flyer,posters, fact sheet, FAQs pocket books)
☐ Other
(Mark all that apply)

Other, please specify ______________________________

Where else did you get information about oral PrEP apart from where you first heard about it?

☐ Clinic
☐ Friends
☐ Colleagues
☐ Social media
☐ Internet
☐ Radio/TV/Newspaper
☐ Printed information (such as flyers,posters,fact sheet, FAQs pocket books)
☐ No other source
☐ Other
(Mark all that apply)

Other, please specify ______________________________

Printed information

What printed information did you see/receive from peer educators or from providers at the facility?	☐ Posters<br>☐ Fact Sheet<br>☐ FAQs<br>☐ PrEP initiation Pack<br>☐ Pocket book<br>☐ Did not receive/see any printed information<br>☐ Other<br>(Mark all that apply)
Other, please specify	__________________________
Which printed information did you find most useful?	◯ Posters<br>◯ Fact Sheet<br>◯ FAQs<br>◯ PrEP initiation Pack<br>◯ Pocket book<br>◯ Did not receive/see any printed information<br>◯ Other<br>(Mark all that apply)
Other, please specify	__________________________
Have you seen the "we are the generation that will end HIV" slogan	◯ Yes<br>◯ No<br>◯ Not sure
What did you think of the "we are the generation that will end HIV" slogan?	◯ I liked it<br>◯ I did not like it<br>◯ Nothing<br>(Show participant slogan)
Why did you feel this way?	☐ The message empowered me<br>☐ I liked the design<br>☐ I did not like the design<br>☐ I did not believe in the message<br>☐ Other<br>(Mark all that apply)
Other, please specify	__________________________
Have you seen the " I have the right to live HIV free/ I have the duty to help stop the spread of HIV" slogan	◯ Yes<br>◯ No<br>◯ Not sure<br>(Show participant slogan)
What did you think of the "I have the right to live HIV free/I have the duty to help stop the spread of HIV" slogan?	◯ I liked it<br>◯ I did not like it<br>◯ Nothing
Why did you feel this way	☐ The message empowered me<br>☐ I liked the design<br>☐ I did not like the design<br>☐ I did not believe in the message<br>☐ Other<br>(Mark all that apply)

Other, please specify

____

Oral Prep Knowledge Heard Of Oral Prep

Record ID ______________________

Now I would like to ask about what you may have heard about oral PrEP. Please answer true or false regarding the following statements. It's okay if you are unsure if the statement is true or false; just tell me "I don't know".

***DO NOT PROMPT RESPONSES!!**

Oral PrEP is prescribed for people who are HIV negative
- ○ True
- ○ False
- ○ I don't know

You must take an HIV test before you can be prescribed oral PrEP.
- ○ True
- ○ False
- ○ I don't know

Condom use is still advised when using oral PrEP.
- ○ True
- ○ False
- ○ I don't know

Oral PrEP has potential side effects
- ○ True
- ○ False
- ○ I don't know

Oral PrEP works best when it is taken daily and according to the instructions.
- ○ True
- ○ False
- ○ I don't know

Perceptions and Beliefs about Oral PrEP

We now would like to ask you what you think and believe about using PrEP.

***DO NOT PROMPT RESPONSES!!**

What do you think are the benefits of using oral PrEP?
- ☐ Prevents HIV infection
- ☐ Prevents other STIs
- ☐ Don't have to use a condom
- ☐ Can use without the partner's knowledge/cooperation
- ☐ I don't know
- ☐ Other

(Mark all that apply)

Other, please specify ______________________

What do you think are the challenges of using oral PrEP?	☐ Not easily accessible<br>☐ Having to take them daily<br>☐ Side effects<br>☐ Stigma<br>☐ I don't know<br>☐ Other<br>(Mark all that apply)
Other, please specify	__________________________________
Do you think oral PrEP is safe?	◯ Yes<br>◯ No<br>◯ I don't know
Do you think oral PrEP is effective for preventing HIV?	◯ Yes<br>◯ No<br>◯ I don't know

ORAL PREP USE PRACTICES

We would like to ask you questions about your use or lack of oral PrEP.

Have you ever been offered oral PrEP before by a health care worker?	◯ Yes<br>◯ No
What has a healthcare provider told you about the oral PrEP?	☐ How to use oral PrEP<br>☐ When to use oral PrEP<br>☐ Safety<br>☐ Side Effects<br>☐ Managing side effects<br>☐ Other
Other, please specify	__________________________________
Have you ever taken oral PrEP before?	◯ Yes<br>◯ No- Never been offered oral PrEP<br>◯ No- I declined<br>◯ No
What was your reason for declining?	◯ Not sexually active<br>◯ Only have one faithful sexual partner<br>◯ Afraid of side effects<br>◯ Clinic is too far<br>◯ My partner did not want me to use it<br>◯ My family did not want me to use it<br>◯ Afraid of stigma<br>◯ I did not feel I was at risk of HIV<br>◯ Other
Other, please specify	__________________________________

If you have taken oral PrEP before, why did you decide to start?	◯ I am sexually active<br>◯ I feel that I am at risk for HIV<br>◯ I have multiple sexual partners<br>◯ I have clients who I believe could be HIV positive<br>◯ I have clients who do not want to use condoms<br>◯ Other
Other, please specify	__________________________________
Which printed information influenced or helped with your decision to use PrEP?	☐ Posters<br>☐ Fact sheet<br>☐ FAQs<br>☐ PrEP initiation packet<br>☐ Pocket book<br>☐ Did not influence my decision<br>☐ Other
Other, please specify	__________________________________
Are you currently taking oral PrEP?	◯ Yes<br>◯ No
Which printed information helped you to continue with your use of PrEP?	☐ Posters<br>☐ Fact sheet<br>☐ FAQs<br>☐ PrEP initiation packet<br>☐ Pocket book<br>☐ Did not help me to continue with my use of PrEP<br>☐ Other
Other, please specify	__________________________________
Which printed information would influence or help you with your decision to start using PrEP in the future?	☐ Posters<br>☐ Fact sheet<br>☐ FAQs<br>☐ PrEP initiation packet<br>☐ Pocket book<br>☐ None will influence my decision<br>☐ Other
Other, please specify	__________________________________
How long have you been taking/had you taken oral PrEP?	◯ 1-2 months<br>◯ 3-5 months<br>◯ 6-8 months<br>◯ 9-12 months<br>◯ More than 12 months<br>◯ Never taken oral PrEP

For Current PrEP users<br><br>If you are still taking oral PrEP, what is your reason for continuing to take it?	☐ I am sexually active<br>☐ I feel that I am at risk for HIV<br>☐ I have multiple sexual partners<br>☐ I have clients who I believe could be HIV positive<br>☐ I have clients who do not want to use condoms<br>☐ Other<br>(Mark all that apply)
Other, please specify	______________________________
For clients who discontinued PrEP only<br><br>If you are not taking PrEP anymore , why did you decide to stop?	☐ No longer sexually active<br>☐ Only have one faithful sexual partner<br>☐ Side effects were too much<br>☐ Clinic is too far<br>☐ Clinic didn't offer PrEP anymore<br>☐ My partner told me to stop using PrEP<br>☐ I felt stigmatized<br>☐ Other<br>(Mark all that apply)
Other, please specify	______________________________
Does/did your main partner (non-sex work partner) know that you are taking oral PrEP?	◯ Yes<br>◯ No (sex worker)<br>◯ No (not sex worker)
If yes, how often do you and your partner use condoms?	◯ The same as before you started PrEP<br>◯ More frequently than before you started PrEP<br>◯ Less frequently than before you started PrEP<br>◯ We have never used condoms
For sex-workers only<br><br>Does/did your clients know that you are taking oral PrEP?	◯ Yes<br>◯ No
For sex workers only - skip if not a sex worker<br><br>If yes, are you able to negotiate condom use with your clients?	◯ Yes,always<br>◯ Yes, sometimes<br>◯ No, not since I started PrEP<br>◯ No, I have never used condoms with my clients
Do /did any referent others (residing with you or not) and/or people living with you know that you are taking oral PrEP?	◯ Yes<br>◯ No
If yes, who knows/knew about your oral PrEP use?	☐ Family Member<br>☐ Child/children<br>☐ Friends<br>☐ House-mates<br>☐ Main Partner/s (non-sex work)<br>☐ Casual Partner/s (non-sex work)<br>☐ Other<br>(Mark all that apply)
Other, please specify	______________________________

Who was supportive of your use of oral PrEP?	☐ Family Member<br>☐ Child/children<br>☐ Friends<br>☐ House-mates<br>☐ Main Partner/s (non-sex work)<br>☐ Casual Partner/s (non-sex work)<br>☐ None of the above<br>☐ Other<br>(Mark all that apply)
Other, please specify	__________________________
What was their reason for not being supportive?	☐ They did not understand why I need to take an ARV if I am negative<br>☐ Did not understand how PrEP works<br>☐ Afraid I would have side effects<br>☐ They did not think that I needed it<br>☐ Other<br>(Mark all that apply)
Other, please specify	__________________________
Is/was it ever difficult for you to take oral PrEP when someone from your family/home can see you	◯ Yes<br>◯ No<br>◯ Sometimes
Is/was there anyone anything that regularly reminds/ reminded you to take your oral PrEP on time?	◯ Yes<br>◯ No<br>◯ Cannot remember
Who or what reminds/ reminded you to take your oral PrEP on time?	◯ I remind myself<br>◯ Family members<br>◯ Friends/colleagues<br>◯ Cellphone reminder<br>◯ Favorite TV/radio show<br>◯ Cannot remember<br>◯ Other
Other, please specify	__________________________
In the past month, have you ever not take your oral PrEP?	◯ Yes<br>◯ No<br>◯ Not applicable- been off PrEP for more than a month

What circumstance led you to miss taking oral PrEP in the past month?	☐ I was sick<br>☐ Alarm didn't go off<br>☐ I wasn't home<br>☐ Did not want people to find out<br>☐ There were people around me<br>☐ Fear of partner not agreeing<br>☐ Prescription finished<br>☐ Tired of side effects<br>☐ Drugs<br>☐ Alcohol<br>☐ Unsure/Forgot<br>☐ Other<br>(Mark all that apply)
Other, please specify	______________________________
How often do you take the drugs per week?	◯ Daily<br>◯ Once<br>◯ Twice<br>◯ more than 3 times
How often do you take alcohol per week	◯ Daily<br>◯ Once<br>◯ Twice<br>◯ more than 3 times
Have you missed a dose in the past 3 days?	◯ Yes<br>◯ No
When over the past 3 days have you missed your prescribed daily dose of PrEP?	☐ Yesterday<br>☐ Day before yesterday<br>☐ 3 days ago<br>☐ Unsure/Cannot remember<br>(Mark all that apply)
What circumstances led you to miss taking oral PrEP over the past 3 days?	☐ I was sick<br>☐ Alarm didn't go off<br>☐ I wasn't home<br>☐ There were people around me<br>☐ Fear of partner not agreeing<br>☐ Prescription finished<br>☐ Tired of side effects<br>☐ Unsure/Forget<br>☐ Other<br>(Mark all that apply)
Other, please specify	______________________________
Is/was it ever difficult for you to take your oral PrEP on weekends?	◯ Yes<br>◯ No<br>◯ Sometimes

What other things make/made it difficult to take all your oral PrEP on time?	☐ Stress<br>☐ Work<br>☐ Family life<br>☐ Going away from home<br>☐ Being too busy<br>☐ Fear of partner opposition<br>☐ Fear of family opposition<br>☐ Did not find it difficult<br>☐ Other<br>(Mark all that apply)
Other, please specify	______________________________
Have you ever taken a double dose of oral PrEP after missing a dose?	○ Yes<br>○ No- did not take a double dose<br>○ No- never missed a dose<br>○ Cannot remember
Thinking about the different methods to prevent HIV, how easy or difficult would you say it is to use oral PrEP successfully?	○ Very easy<br>○ Moderately easy<br>○ Moderately difficult<br>○ Very difficult
How easy or difficult has it been for you to use both oral PrEP and condoms with your main partner (non-sex work)?	○ Very easy<br>○ Moderately easy<br>○ Moderately difficult<br>○ Very difficult
For sex workers only<br><br>How easy or difficult has it been for you to use both oral PrEP and condoms with your clients?	○ Very easy<br>○ Moderately easy<br>○ Moderately difficult<br>○ Very difficult<br>○ Not applicable
Thinking about both condoms and oral PrEP, which method would you say works better for you	○ Condoms<br>○ Oral PrEP<br>○ Both<br>○ Neither

Oral Prep Beliefsexperience

Record ID ______________________

BELIEFS/EXPERIENCE WITH SIDE EFFECTS OF PREP
We would like to ask you some questions about beliefs and experiences you have had with side effects when using PrEP

***DO NOT PROMPT RESPONSES!!**

Do you believe that there are side effects to oral PrEP use?
- ◯ Yes
- ◯ No
- ◯ I don't know

What side effects do you think are associated with oral PrEP use?
- ☐ Nausea
- ☐ Vomiting
- ☐ Stomach pain
- ☐ diarrhea
- ☐ headache
- ☐ dizziness
- ☐ depression
- ☐ joint pain
- ☐ trouble sleeping
- ☐ strange dreams
- ☐ back pain
- ☐ itching or skin rash
- ☐ Changes in the color of skin on your palms or soles of your feet
- ☐ Changes in the shape or location of body fat (especially in your arms, legs, face, neck, breasts and waist)
- ☐ Upset stomach
- ☐ Loss of appetite
- ☐ Weight gain
- ☐ I don't know
- ☐ Other

(Mark all that apply)

Other, please specify ______________________

Have you experienced any side effects since you started taking oral PrEP?
- ◯ Yes
- ◯ No
- ◯ Not applicable

Which ones?	☐ Nausea<br>☐ Vomiting<br>☐ Stomach pain<br>☐ diarrhea<br>☐ headache<br>☐ dizziness<br>☐ depression<br>☐ joint pain<br>☐ trouble sleeping<br>☐ strange dreams<br>☐ back pain<br>☐ itching or skin rash<br>☐ Changes in the color of skin on your palms or soles of your feet<br>☐ Changes in the shape or location of body fat (especially in your arms, legs, face, neck, breasts and waist)<br>☐ Upset stomach<br>☐ Loss of appetite<br>☐ Weight gain<br>☐ I don't know<br>☐ Other<br>(Mark all that apply)
Other, please specify	______________________________
Would you consider these side effects tolerable?	◯ Yes<br>◯ No
Which side effects were not tolerable?	☐ Nausea<br>☐ Vomiting<br>☐ Stomach pain<br>☐ diarrhea<br>☐ headache<br>☐ dizziness<br>☐ depression<br>☐ joint pain<br>☐ trouble sleeping<br>☐ strange dreams<br>☐ back pain<br>☐ itching or skin rash<br>☐ Changes in the color of skin on your palms or soles of your feet<br>☐ Changes in the shape or location of body fat (especially in your arms, legs, face, neck, breasts and waist)<br>☐ Upset stomach<br>☐ Loss of appetite<br>☐ Weight gain<br>☐ I don't know<br>☐ Other
Other	______________________________

Did these side effects affect your daily life (work, etc?)	◯ Yes<br>◯ No<br>(Interviewer to ask participant before moving to the next section: Are you happy to continue with the survey, do you have any questions at this time?)

SATISFACTION WITH ORAL PREP SERVICES (INCLUDING WAIT TIME AND INTERACTION WITH PROVIDERS)

This is the last section of the questionnaire. Thank you for staying with me!. We would now like to ask you about the health service you receive PrEP at and if you are satisfied with this service.

***DO NOT PROMPT RESPONSES!!**

How long have you been attending this clinic?<br><br>(Note to Interviewer: months)	________________________________
Before today, how often did you visit this clinic in the past 6 months?	◯ Have not visited the clinic in the past 6 months<br>◯ Once<br>◯ Twice<br>◯ More than 3 times
What were the reasons for your visits?	☐ Accessing medication<br>☐ Accessing contraception<br>☐ Routine check up<br>☐ Illness<br>☐ Injury<br>☐ Personal reasons<br>☐ Other<br>(Mark all that apply)
Other, please specify	________________________________
How long does it take you to travel to this clinic (using the mode of transportation usually taken)?	◯ Less than 15 minutes<br>◯ 15-30 minutes<br>◯ 30-60 minutes<br>◯ Over an hour
How do you feel about the quality of care you receive in this clinic?	◯ It's great<br>◯ It's okay<br>◯ They can do better<br>◯ It's bad
How do you feel about the waiting time at this clinic?	◯ Acceptable<br>◯ Too long

In general, how do providers in this clinic treat you as a person?	☐ They treat me well<br>☐ They are very helpful<br>☐ They are rude to me<br>☐ They are judgmental toward me<br>☐ They do not want to help me<br>☐ Other<br>(Mark all that apply)
Other, please specify	__________________________
How often do providers explain things to you in a way that is easy to understand?	◯ Always<br>◯ Most times<br>◯ Sometimes<br>◯ Never
How often do providers listen carefully to you?	◯ Always<br>◯ Most times<br>◯ Sometimes<br>◯ Never
How often do providers spend enough time with you?	◯ Always<br>◯ Most times<br>◯ Sometimes<br>◯ Never
How often do you have enough privacy during your clinic visits?	◯ Always<br>◯ Most times<br>◯ Sometimes<br>◯ Never
Do you feel providers gave you the choice of whether or not to use PrEP?	◯ Yes<br>◯ No (never user)<br>◯ No, (current/past)<br>◯ Cannot remember
In respect of receiving oral PrEP from providers at the facility, were you satisfied with the service received?	◯ Yes<br>◯ No<br>◯ Somewhat
If no or somewhat, why were you not satisfied?	◯ Waiting time too long<br>◯ Provider was rude<br>◯ Provider was unfriendly<br>◯ Provider was judgmental towards me<br>◯ Provider did not answer my questions<br>◯ I did not have enough time with the provider<br>◯ Other
Other, please specify	__________________________
In your opinion, since the time oral PrEP has been available at this facility, do you feel other services you receive have been affected (i.e: in terms of waiting time, availability of provider, etc.)	◯ Yes-positively<br>◯ Yes-negatively<br>◯ No (End of survey)<br>◯ I am not sure (End of survey)

IF its end of survey: Interviewer to address participant before concluding survey: Do you have any questions at this time?

Thank you for taking the time to complete this survey

Which services has it affected?	☐ Family Planning<br>☐ STI treatment<br>☐ ART provision<br>☐ Other<br>(Mark all that apply)
Other, please specify	______________________
If it has negatively affected services, how so?<br><br>(Note to interviewer before concluding survey ask: Do you have any questions at this time?)<br><br>Thank you for taking the time to complete this survey	◯ Longer waiting times<br>◯ Not enough time with providers<br>◯ Providers are in a rush<br>◯ Other
Other, please specify<br><br>(Note to interviewer before concluding survey ask: Do you have any questions at this time?)<br><br>Thank you for taking the time to complete this survey	____________________________
Name of data collector/ interviewer	______________________
