# Supplementary material for: Factors influencing uptake, continuation, and discontinuation of oral PrEP among clients at sex worker and MSM facilities in South Africa
Source: PLoS One. 2020 Apr 30;15(4):e0228620. doi: 10.1371/journal.pone.0228620 (PMC7192496; doi:10.1371/journal.pone.0228620)
Supplement: S3 Appendix — (PDF) [file pone.0228620.s004.pdf]

ACCESS - PrEP NEVER Users IDI Guide

Primary research questions:

1. **For people who have heard about PrEP but have never used it, why haven't they used it?**
2. **What other information around oral PrEP do never users need, and what additional information can clinics and NDoH provide?**

**Introduction**	
My name is ________________________________. Thank you for agreeing to take some time to talk to us today about oral PrEP for HIV prevention. I am representing research organisations who are collecting data on behalf of the National Department of Health. I would like to confirm with you if your name is ____________. I would like to confirm with you if you have never used PrEP?<br><br>This interview is a follow up to the interview/survey you completed in (month and year) ____________________. Through this discussion, we would like to discuss certain, more specific topics on oral PrEP that we hope will help in improving the overall delivery of oral PrEP in South Africa.<br><br>I am looking forward to hearing your thoughts on the questions I will ask you. Please know there are no right or wrong answers or opinions about the topics we are discussing, so feel free to share your thoughts openly. We're simply looking to collect opinions, experiences, and beliefs from a range of men and women who participated in the earlier phase of this study. Also, feel free to skip any questions that you do not want to answer.	
**Section A: Biography**	
1. Please tell me a brief history of yourself to help me get to know you better.	*Guiding probes: where they grew up, what they do for a living, if they have family, are they new to the area, any other details that would help us to know you better. [Note: no need to ask all probes, keep this section fairly short]*
2. What are your thoughts about HIV prevention?	
3. Do you feel that you might be at risk of HIV infection?	*[if yes] What situations in your life put you at risk of HIV?*
**Section C: Sexual behaviours and HIV Prevention Practices**	
4. What do you do to protect yourself from getting HIV?	*a. What types of protection, if any, do you use? [Examples: condoms, traditional herbs or methods, abstinence]*<br>*b. [If any types mentioned] How regularly do you use this protection?*

**Section D: HIV Prevention Practices**	
5. What have been your biggest challenges to using HIV prevention methods regularly?	*[If no challenges mentioned, skip to question 6]*<br>a. *Have there been any times when you have had unprotected sex with a partner whose HIV status you don't know?*<br>b. *What do you do to manage these challenges or seek help for them? [Probe: Clinic, other sources]*
6. What have you heard about oral PrEP?	*[If no skip to question 7]*<br>a. *Where did you hear this?*<br>b. *Has a healthcare provider ever offered PrEP to you? [If yes] Tell me about that.*<br>c. *Have you ever talked to someone currently taking PrEP about their experiences?*<br>d. *Have these interactions made you more interested or less interested in taking PrEP yourself? Please describe*
7. Would you ever consider using oral PrEP to protect yourself from HIV infection?	a. *What are the main reasons you would or wouldn't use it?*<br>b. *[Probe for stigma if not mentioned]*
8. Oral PrEP contains the same medication that is used for treating HIV-positive people. What do you think about taking a medication that is also used for HIV treatment?	*[guiding probe]*<br>a. *Why do you say so*
9. I'd like to ask you about violence you may have experienced from sexual partners. I know that violence or abuse can be difficult to speak about so it is up to you whether you want to answer these questions. Can you tell me about any violence you've experienced from sexual partners in your personal life or work life?	*[If no personal violence mentioned, skip to probe c]*<br>a. *Does this violence affect your ability to use particular HIV prevention methods?*<br>b. *[If yes] In what ways?*<br>c. *What about your friends or people in your circle that might have experienced violence from sexual partners?*
**Section E: Information, Education and Communication**	
Transition statement: Thank you for your responses. Now I would like to ask you a few questions about the printed materials giving information about PrEP that you may have seen in this clinic or have received from peer educators.	
10. Have you ever seen the printed information materials about oral PrEP?	*[If answer is no, skip to Question 14.]*
11. What do you think about the printed information materials?	a. *[Probe: Posters, flyer, frequently asked question brochure, pocket book, etc.]*<br>b. *How did you feel when you saw the "we are the generation that will end HIV" image?*<br>c. *What do you think of sharing the materials with your friends?*

12. What, if anything could make these materials more useful?	*a. If you do, please can you explain how you think they can be improved on?*<br>*b. Is there any extra information that you feel you need? If so what information?*
13. Other than these materials (NOTE to interviewer: display page with printed materials) where else have you heard about oral PrEP?	*a. What did you learn from that source?*<br>*b. Was this information helpful?*
**Section F: Satisfaction with service**	
14. How satisfied do you feel about the service you receive from this clinic?	*a. What services are you accessing today?*<br>*b. What other services do you access from this clinic?*<br>*c. How do providers interact with you? [Probe: do they listen to you, how do they treat you]*<br>*d. How do you feel the service at this clinic compares to a public clinic?*
**General Comments**	
15. Do you have anything you would like to tell me?	

ACCESS - PrEP CURRENT Users IDI Guide

Primary research questions:

1. **What are the reasons that people discontinue PrEP? Cycle on and off?**
2. **What are challenges around adherence and consistent pill-taking?**
3. **What other information around oral PrEP do current users need, and what additional information can clinics and NDoH provide?**

**Introduction**	
My name is ______________________________. Thank you for agreeing to take some time to talk to us today about oral PrEP for HIV prevention. I am representing research organisations who are collecting data on behalf of the National Department of Health. I would like to confirm with you if your name is ____________. Are you a current PrEP user?<br><br>This interview is a follow up to the interview/survey you completed in (month and year) ____________________. Through this discussion, we would like to discuss certain, more specific topics on oral PrEP that we hope will help in improving the overall delivery of oral PrEP in South Africa.<br><br>I am looking forward to hearing your thoughts on the questions I will ask you. Please know there are no right or wrong answers or opinions about the topics we are discussing, so feel free to share your thoughts openly. We're simply looking to collect opinions, experiences, and beliefs from a range of men and women who participated in the earlier phase of this study. Also, feel free to skip any questions that you do not want to answer	
**Section A: Biography**	
1. Please tell me a brief history of yourself to help me get to know you better.	*Guiding probes: where they grew up, what they do for a living, if they have family, are they new to the area, any other details that would help us to know you better. [Note: no need to ask all probes, keep this section fairly short]*
**Section B: PrEP decision-making and disclosure**<br>**Now I would like to talk to you about using oral PrEP.**	
2. Tell me about your decision to start using oral PrEP.	*a. Is there anything else that influenced your decision to start using PrEP?*<br>*b. How long have you been using PrEP?*<br>c. *What situations in your life put you at risk of HIV?*
3. Who (if anyone) have you told about your PrEP use?	a. *Has telling those people made it easier or harder to use PrEP? In what ways?*<br>b. *What kinds of support have they provided? [If no response, probe for general encouragement, transport to clinic, reminders to use PrEP, help dealing with side effects]*<br>c. *What, if any, other kinds of support would you like?*<br>d. *Who haven't you told about your PrEP use?*<br>e. *What are the reasons you haven't told them?*<br>f. *Is there anyone you would never tell?*

	g. *What are the reasons you will never tell them.*
**Section C: Oral PrEP Practices**	
4. Have you ever heard of instances where people share their oral PrEP with others?	a. *Have you ever shared your oral PrEP with anyone? [If never shared, skip to question 5]*<br>b. *Tell me more about this.*<br>c. *Who did you share your PrEP with?*<br>d. *What were the reasons you shared it?*
5. What have been your biggest challenges using PrEP?	*[If no challenges mentioned, skip to question 6]*<br>*a. [Probe for stigma if not mentioned]*<br>*b. How have you dealt with these challenges? [probe: clinic, other sources of health]*<br>*c. Do you ever miss a dose because of these challenges?*
6. Please describe any time you have stopped taking PrEP for a while and then started taking PrEP again.	*[If they have never done this, skip to question 7]*<br>*a. Why did you stop taking PrEP?*<br>*b. What made you start taking it again?*<br>*c. How many times have you stopped and started?*<br>d. *What additional support do you think would have helped you stay on PrEP during these times?*
7. Many people have tried PrEP but then stopped using it. What helps you keep using it?	*a. What additional support would help you keep using it?*<br>*b. Can you share some methods that has worked for you to keep using PrEP?*
8. Please describe what it's like trying to take oral PrEP every day.	*a. What challenges have you faced?*<br>*b. What has helped you to take it every day?*<br>*c. Who (if anyone) have you asked for help so you can take your pill every day?*<br>*d. If you have ever gone to the clinic for help taking PrEP every day, have you found this to be helpful?*
9. What side effects have you experienced from taking PrEP?	*[If no personal side effects, ask probe H]*<br>*a. How long did you use oral PrEP before you started experiencing side effects?*<br>*b. How have these side effects affected your daily life?*<br>*c. How have you dealt with them?*<br>*d. How long did these side effects last?*<br>*e. Were you taking any other medication along with your oral PrEP at the time?*<br>*f. If you have ever gone to the clinic for help with side effects, did you find this helpful?*<br>***g.*** *[If they have never gone to the clinic] Why did you not go to the clinic for help with side effects?*

	**h.** *Are there any side effects that you have heard from other people that are taking PrEP?*
10. Oral PrEP contains the same medication that is used for treating HIV-positive people. What do you think about taking a medication that is also used for HIV treatment?	*[guiding probe]*<br>a. *Why do you say so?*
11. I'd like to ask you about violence you may have experienced from sexual partners. I know that violence or abuse can be difficult to speak about so it is up to you whether you want to answer these questions. Can you tell me about any violence you've experienced from sexual partners in your personal life or work life?	*[if no personal violence mentioned, skip to probe f]*<br>a. *Were you using oral PrEP at the time that this violence occurred?*<br>b. *[if yes] Do you think what you experienced was because you were using oral PrEP?*<br>c. *Tell me the reasons you think that.*<br>d. *Do you think this violence affects your ability take oral PrEP?*<br>e. *[If yes] In what ways?*<br>f. *What about your friends or people in your circle that might have experienced violence from sexual partners?*
**Section D: Satisfaction with oral PrEP service**	
12. How satisfied do you feel with the service you receive from this clinic?	a. *How do providers interact with you? [Probe: do they listen to you, how do they treat you]*<br>b. *How do you feel the service at this clinic compares to a public clinic?*
13. Please describe what role your provider had in your decision to go on PrEP.	a. *Have you ever felt pressured to take PrEP against your will?*<br>b. *If so, what happened?*<br>c. *Do you think you knew enough about oral PrEP and what it was before you started taking it?*
**Section E: Information, Education and Communication**	
Transition statement: Thank you for your responses. Now I would like to ask you a few questions about the printed materials giving information about PrEP that you may have seen in this clinic or have received from peer educators.	
14. What do you think about the printed information materials?	a. *[Probe: Posters, flyer, frequently asked question brochure, pocket book, etc.]*<br>b. *How did you feel when you saw the "*we are the generation that will end HIV" *image/ picture?*<br>c. *What was your reaction to it?*<br>**d.** *What do you think of sharing the materials with your friends?*
15. Please tell me what role did these materials have in your decision to take PrEP?	
16. What, if anything could make these materials more useful?	a. *If you do, please can you explain how you think they can be improved on?*

	b. Is there any extra information that you feel you need? If so what information?
17. Other than these materials (NOTE to interviewer: display page with printed materials) where else have you heard about oral PrEP?	*a. What did you learn from that source?*<br>*b. Was this information helpful?*
**General Comments**	
18. Do you have anything else about your experiences taking PrEP that you would like to share with me?	

ACCESS – PAST PrEP Users IDI Guide

Primary research questions:

1. **What are the reasons that people discontinue PrEP? Cycle on and off?**
2. **What are challenges around adherence and consistent pill-taking?**
3. **What additional information do clients need regarding oral PrEP, and what can clinics and NDoH do to fill these gaps?**

**Introduction**	
My name is _______________________________. Thank you for agreeing to take some time to talk to us today about oral PrEP for HIV prevention. I am representing research organisations who are collecting data on behalf of the National Department of Health. I would like to confirm with you if your name is ____________. I would like to confirm with you that you have used PrEP in the past but do not currently use it.<br><br>This interview is a follow up to the interview/survey you completed in (month and year) ____________________. Through this discussion, we would like to delve into certain, more specific topics on oral PrEP that we hope will help in improving the overall delivery of oral PrEP in South Africa.<br><br>I am looking forward to hearing your thoughts on the questions I will ask you. Please know there are no right or wrong answers or opinions about the topics we are discussing, so feel free to share your thoughts openly. We're simply looking to collect opinions, experiences, and beliefs from a range of women who participated in the earlier phase of this study. Also, feel free to skip any questions that you do not want to answer.	
**Section A: Biography**	
1. Please tell me a brief history of yourself to help me get to know you better.	*Guiding probes: where they grew up, what they do for a living, if they have family, are they new to the area, any other details that would help us to know you better. [Note: no need to ask all probes, keep this section fairly short]*
**Section B: PrEP decision-making and disclosure**	
Transition statement: Now I would like to talk to you about using PrEP.	
2. Tell me about your decision to start using oral PrEP.	a. *How long did you take PrEP?*<br>b. *What were the reasons you thought it was necessary for you to take PrEP?*
3. Tell me about your experience taking PrEP.	
4. When you were using oral PrEP, who (if anyone) did you tell about your PrEP use?	a. *Did telling those people make it easier or harder to use oral PrEP? In what ways?*<br>b. *What kinds of support did they provide? [If no response, probe for general encouragement, transport to clinic,*

	reminders to use PrEP, help dealing with side effects]<br>c. *What, if any, other kinds of support would you have liked?*<br>d. *Who did you not tell about your PrEP use?*<br>**e.** *What are the reasons you did not tell them?*
**Section C: Oral PrEP Practices**	
5. Have you ever heard of instances where people share their oral PrEP with others?	a. *Did you ever share your oral PrEP with anyone?*<br>*[If never shared, skip to question 6]*<br>b. *Who did you share your PrEP with?*<br>c. *What were the reasons you shared it?*
6. When you were taking PrEP, what were your biggest challenges using it?	*[If no challenges mentioned, skip to probe D]*<br>a. *[Probe for stigma if not mentioned]*<br>b. *Did you ever miss a dose because of these challenges?*<br>c. *What did you do to manage these challenges or seek help for them? [probe: clinic, other sources]*<br>d. *What have you heard are other people's biggest challenges using PrEP?*
7. Please describe any time you stopped taking PrEP for a while and then started taking PrEP again.	*[If they have never done this, skip to question 8]*<br>a. *What made you stop taking PrEP?*<br>b. *What made you start taking it again?*<br>c. *How many times did you stop and start?*
8. Please describe what it was like trying to take oral PrEP every day.	a. *What challenges did you face?*<br>b. *What helped you take it every day?*<br>c. *Who (if anyone) did you ask for help so you could take your pill every day?*<br>d. *If you ever went to the clinic for help taking PrEP every day, did you find it to be helpful?*
9. What side effects did you experience when you were taking PrEP?	*If no personal side effects, ask probe H]*<br>*If yes, tell me more about them*<br>a. *How long did you use oral PrEP before you started experiencing side effects?*<br>b. *How have these side effects affected your daily life?*<br>c. *How have you dealt with them?*<br>d. *How long did these side effects last?*<br>e. *Were you taking any other medication along with your oral PrEP at the time?*<br>f. *If you have ever gone to the clinic for help with side effects, did you find this helpful?*<br>***g.*** *[If they have never gone to the clinic] Why did you not go to the clinic for help with side effects?*

	h. *Are there any side effects that you have heard from other people that are taking PrEP?*
10. Oral PrEP contains the same medication that is used for treating HIV-positive people. What did you think about taking a medication that was also used for HIV treatment?	*a. Why do you say so?*
11. Since you are no longer on PrEP, please tell me about your decision to stop using PrEP.	*a. What were the main reasons that you stopped taking oral PrEP?*<br>*b. [Probe for stigma if not mentioned]*<br>*c. Please describe whether or not you felt that PrEP met your needs.*
12. Do you think you'll use PrEP again in the future?	*a. What are the main reasons you would or wouldn't use it again?*
13. I'd like to ask you about violence you may have experienced from sexual partners. I know that violence or abuse can be difficult to speak about so it is up to you whether you want to answer these questions. Can you tell me about any violence you've experienced from sexual partners in your personal life or work life?	*[If no personal violence mentioned, skip to probe f]*<br>*a. Were you using oral PrEP at the time that this violence occurred?*<br>*b. [If yes] Do you think what you experienced was because you were using oral PrEP?*<br>*c. Tell me the reasons you think that.*<br>*d. Did this violence affect your ability to take oral PrEP?*<br>e. *[If yes] In what ways?*<br>f. *What about your friends or people in your circle that might have experienced violence from sexual partners?*
**Section D: Satisfaction with oral PrEP service**	
**14.** How satisfied did you feel with the service you received from the clinic where you got PrEP?	*a. How did providers interact with you? [Probe: did they listen to you, how did they treat you]*<br>*b. Did you feel like the service at this clinic is better or worse than at a public clinic?*
15. Please describe what role your provider had in your decision to go on PrEP.	*a. Please describe if you ever felt pressured to take PrEP against your will.*<br>*b. Do you feel like you knew enough about oral PrEP and what it was before you started taking it?*
**Section E: Information, Education and Communication**	
Transition statement: Thank you for your responses. Now I would like to ask you a few questions about the printed materials giving information about PrEP that you may have seen in this clinic or have received from peer educators.	
16. What do you think about the printed information materials?	*a. [Probe: Posters, flyer, frequently asked question brochure, pocket book, etc.]*<br>*b. How did you feel when you saw the "*we are the generation that will end HIV" *image/picture?*<br>*c. What was your reaction to it?*

	**d.** *What do you think of sharing the materials with your friends?*
17. Please tell me what role did these materials have in your decision to take PrEP?	
18. What, if anything could make these materials more useful?	*a. If you do, please can you explain how you think they can be improved on?*<br>*b. Is there any extra information that you feel you need? If so what information?*
19. Other than these materials (NOTE to interviewer: display page with printed materials) where else have you heard about oral PrEP?	*a. What did you learn from that source?*<br>*b. Was this information helpful?*
**General Comments**	
20. Do you have anything else about your experiences taking PrEP that you would like to tell me?	
